# Patterns of genetic structure and evidence of Egyptian Citrus rootstock based on informative SSR, LTR-IRAP and LTR-REMAP molecular markers

**DOI:** 10.1186/s43141-021-00128-z

**Published:** 2021-02-10

**Authors:** Mohamed Abd S. El zayat, Ahmed H. Hassan, Elsayed Nishawy, Mohammed Ali, Mohamed Hamdy Amar

**Affiliations:** Egyptian Deserts Gene Bank, Desert Research Center, Cairo, Egypt

**Keywords:** *Citrus*, Microsatellite, Retrotransposons, Structure analysis, PCoA

## Abstract

**Background:**

Releasing the draft genome of sweet orange provides useful information on genetic structure and molecular marker association with heritable breeding traits in citrus species and their structures. Last decades, microsatellite and retrotransposons are well known as a significant diverse component of the structural evolution. They represented the most potent elements for assessing sustainable utilization of the complicated classification in citrus breeding. Our study was performed to verify the structure analysis and the parentage genetic diversity among the Egyptian citrus rootstocks and the related species.

**Results:**

Here, the performance of 26 SSR and 14 LTR-IRAP in addition to 20 LTR-REMAP markers have been used to conduct the discriminating power and the status of the genetic structure analysis among twenty specimens of citrus genotypes. As a result, the three markers approach exhibited a remarkable variation among the tested genotypes. Overall, the three markers have different discrimination power; the co-dominant SSR markers can differentiate within the group level only in addition to the species level of sour orange, while the dominant markers LTR-IRAP had the ability to discriminate among the group level in addition to species level and the origin of acids. Similarly, LTR-REMAP is suitable for classifying the group level and species level for mandarins as well the origin of Egyptian acids; probably due to it is integration of SSR and LTR-IRAP techniques. Structure and PCoA results of LTR-REMAP marker in strong support for the group structure of citrus species have been divided into four sets: acids, grapefruit/pummelo, mandarin/orange, and sour orange.

**Conclusion:**

Our findings of the genetic structure analysis support the monophyletic nature of the citrus species; are able to provide unambiguous identification and disposition of true species and related hybrids like lemon, lime, citron, sour orange, grapefruit, mandarin, sweet orange, pummelo, and fortunella; and resulted in their placement in individual or overlap groups. The outcomes of these results will offer helpful and potential information for breeding programs and conservation approaches as a key stage toward identifying the interspecific admixture and the inferred structure origins of Egyptian citrus rootstock and acid cultivars.

## Background

The release of the several plant genomes platform in the last decade enriched the genetic structure, molecular marker-assisted breeding, and the basis of molecular biological research, where the genomics data in citrus have accumulated rapidly after the release of the sweet orange draft genome [1]. Presently, the first draft genome of citrus provides valuable information resources for understanding and development of several repeated sequences approaches such as microsatellites and retrotransposons regions.

Microsatellites became the marker of choice in plant genetics and breeding research in citrus species [2–6]. In the last decades, the publicly available of citrus EST and BAC-end sequences provides promising information to detect SSR motif for the development of a large number of effective SSR markers; besides, it allows the linkage of heritage traits connected to genomic divergence in citrus germplasm [7].

As it is well known, plants contain extended repetitive elements, many of which are mobile genetic elements (transposons) that are capable of changing their position within the genome. Among these transposable elements, the retrotransposons constitute the largest group and are further grouped into two main classes depending on the presence or absence of flanking long terminal direct repeats (LTR) [2]. These LTR are highly conserved and are exploited for primer design in the development of retrotransposons-based markers [3]. Several retrotransposon-based marker systems have been developed like, inter-retrotransposon amplified polymorphism (IRAP) and retrotransposon-microsatellite amplified polymorphism (REMAP) [4].

The IRAP is a promising marker due to its ability to detect the insertion polymorphisms by amplifying the portion of DNA between two retrotransposons [8, 9]. However, the REMAP is similar to IRAP, but one of the two primers matches a microsatellite motif [10, 11]. In LTR-REMAP, anchor nucleotides are used at the three ends of the simple sequence repeat primer to avoid slippage of the primer between the individual simple sequence repeat motifs [9]. An anchored primer also prevents the detection of variation in repeat numbers within the microsatellite [12]. Abundant in most genomes, microsatellites, or SSR motifs seem to associate with retrotransposons and caused high mutation rates due to polymerase slippage. Therefore, they may be more effectiveness loci for genetic diversity, structure analysis, phylogeny, and plant genotyping within a species or sub-species [13, 14].

Citrus classification is complicated and confusing by many features, such as a long history of cultivation, bud mutation, nuclear embryonic, and a broad cross-compatibility between species and invasive species [15]. The most widely known taxonomic systems for citrus are Swingle [16] and Tanaka [17], who documented 16 and 162 species. Sweet orange, mandarin, pummelo, grapefruit, lemon, lime, and various hybrids are among the most commonly grown and economically important fruit tree crops in the genus citrus [18]. Hence, the development of citrus cultivar through conventional methods is quite problematic, ineffective, expensive, and time-consuming due to its prolonging juvenility, unusual sexual behavior, and complicated genetic background [19]. At present, the number of species to be recognized in citrus and the relationships among genotypes is the major problems in citrus classification [20].

Additionally, root stock has a powerful impact on yield, fruit quality, tree circumference and shape, beside that can also offer tolerance of biotic and abiotic stresses. In Egypt, several valid species or natural hybrids have served as highly effective root stocks involving sour orange, volkamer lemon, Egyptian lime, rough, and eureka lemon. These made citrus rootstock breeding a vital research activity globally. Therefore, the performance of the new species in Egypt should be considered before cultivating to preserve the citrus genetic resources against the invasive citrus species.

Conversely, genetic structure analysis and molecular evolution represent the most powerful tools for evaluating genomes and enabling the association of heritable traits with underlying genomic variation [21]. However, the enhanced performance of DNA markers and their transferability to present a broad presence of varieties also helped in revealing the confused genealogy of native citrus varieties and its origins [22]. Unfortunately, retrotransposons and microsatellites-based markers are still less explored in citrus research comparing to other plant species like *Oryza*, *Triticum*, and *Brassica* [23].

The present study, based on an extensive sampling from China and Egypt, measures structure analysis and the genetic diversity of Egyptian rootstocks and domesticated citrus species with its related. Through this research, the performance of the SSR, LTR-IRAP, and LTR-REMAP markers has been made to conduct the discriminating power and the status of genetic structure and phylogeny of the individual marker in the genus citrus and related species. This data will allow calculation of genetic diversity and the genetic structure within various Egyptian and Chinese citrus species, which has not been previously reported.

## Methods

### Plant materials and genomic DNA isolation (gDNA)

Twenty genotypes belong to the genus citrus and its relative species involved the following major groups of citrus as listed in Table 1. The samples were collected from the National Research Center, Egypt, and for the National Center of citrus Breeding (NCCB), Huazhong Agricultural University (HZAU), Wuhan, China. Genomic DNA of citrus species was extracted from the fresh leaves following the procedure as previously described elsewhere [24]. The quality and concentration of the DNA samples were checked using UV-1601 spectrophotometer (Shimadzu, Japan) and a sub-aliquot of the DNA was subsequently diluted to 50 ng/μl. Both the stock and diluted portions were stored at − 20 °C.

**Table 1:** The accession list of 20 citrus genotypes and its relatives used with SSR, LTR-IRAP and LTR-REMAP markers

Sample No.	Genotype name	Scientific name	Type of fruit	Group name
1	Fingered Citron	*Citrus medica var sarcodactylis*		Citron
2	Eureka Lemon	*Citrus limon (L.)* Burm. f.	Acids	Lemon
3	Egyptian Eureka lemon	*Citrus limon*		Lemon
4	Rough lemon	*Citrus limon (L.)* Burm. f.		Lemon
5	Volkamer lemon	*Citrus volkameriana*		Lemon
6	Egyptian lime	*Citrus aurantifolia (Christm.)* Swingle		Sour lime
7	HB pummelo	*Citrus maxima*	Grapefruits and pummelos	Pummelo
8	Shatian pummelo	*Citrus maxima*		Pummelo
9	Guan Xi Miyon pummelo	*Citrus maxima*		Pummelo
10	Red Marsh grapefruit	*Citrus paradisi* Macfad		Grapefruit
11	Ponkan	*Citrus reticulata* Blanco	Mandarins	Mandarin
12	Guoqing	*Citrus reticulata* Blanco		Mandarin
13	Murcott	*Citrus reticulata* Blanco		Mandarin
14	Jincheng	*Citrus sinensis* Osbeck	Oranges	Sweet orange
15	Valencia	*Citrus sinensis* Osbeck		Sweet orange
16	Anliu	*Citrus sinensis* Osbeck		Sweet orange
17	Cara Cara	*Citrus sinensis* Osbeck		Navel orange
18	Daidai	*Citrus aurantium* L.		Sour orange
19	Bitter orange	*Citrus aurantium* L.		Sour orange
20	Meiwa kumquat	*Fortunella crassifolia* Swingle	Kumquat	Kumquat

### SSR analysis

Twenty-six successful SSR primers were designed from the flanking sequences, using SSRLocatorI V1.1 software [25] according to the sweet orange draft genome and the publicly available of EST and BAC-end sequences in the citrus database (Table 2). PCR amplification reaction was prepared according to the previously described by Amar et al. [26] with minor modifications. PCR mix were performed as follows: in 20 μl of reaction mixture containing 2 μl 10× PCR buffer, 2 mmol MgCl_2_, 200 mmol dNTP, 4 pmol of each primer (forward and reverse), 50 ng template DNA and 1 U Taq DNA polymerase (Ferments). The amplification reaction procedure was as follows: after denaturation at 94 °C for 4 min, the reaction mixture was subjected to amplification for 10 cycles consisting of 30 s at 94 °C, 30 s at 66 °C, and 45 s at 72 °C, followed by 30 cycles consisting of 30 s at 94 °C, 30 s at 55 °C, and 45 s at 72 °C with a final extension of 72 °C for 10 min. The amplification products were separated by 6% poly acrylamide gel electrophoresis (PAGE) and visualized by a simplified silver staining method previously described by Xu et al. [27].

**Table 2:** List of SSR, LTR-IRAP, and LTR-REMAP primers sequence and its optimum annealing temperature

**A: SSR**			
**Primer name**	**Forward**	**Reverse**	**TM**
SA1	TGTATCCCTGCCGTTTCTTC	GAAACTTCCCACTTCGCTCA	57
SA2	ACTTGGGGCTTTCTCACGTT	TTTGCCAGATATTGCTGCTG	57
SA3	TCTCCGAACTCTCGCACTAAA	GGGGGATGTTGGAGATTTTT	58
SA4	CAGTCGATTGTTTGCTGTGG	TTCGGAAATTTTTCTGTGGA	57
SA5	CCACCACTCAATTTTGCTGA	GCATTCACACGATCCACATC	55
SA6	TCTCCGAACTCTCGCACTAAA	GGGGGATGTTGGAGATTTTT	58
SA7	GAGAGAGGTGGCAATTGAGC	TTGCCTCACAACAAACAAAGA	59
SA8	TCACAAATTTATGCCTTGCG	TCGATAGTGCACCACGACAT	53
SA9	TCGAGAAAATTAAGTCTTTTCTTCC	ATTCTTCGGTTCTTGGGCTT	55
SA10	CCCAGGTTAGCAACTTCGTT	CAAAGTCAATTGGAATCTCCTTG	57
SA11	AGCCTTGGCTGAGCTGTAAA	GGGTGCCATTTAAAAACCCT	57
SA12	CCGCCAGATTTTTCATTTTC	GAATCCGCCACCAATTTAAC	53
SA13	AAGAGCACTTGCCGAGGATA	GAATCCCATTTGATCCGAGA	57
SA14	CCAAGTTTTGCTTCCCTTGA	AGCTCTGGTGGATTTCCTGA	55
SA15	TCGAAGAGAGGGAGGAGTCA	AGAACCACCCCCTTCTTTGT	59
SA16	CGGATGGAAGAAAACCTGAA	AGTCGAATTACGGGTTGCAG	55
SA17	GCAGCCCTCAACATGATACA	GCCGTCAACTTTCTTGCTTC	57
SA18	TCTCCTCTCCTCTTGTTCTTCTTC	TTGATGGTCTTGGAAGGGTC	60
SA19	CGCTGAGAACTGAGAAGGAAA	TGCAATTCGATGTTGTTCTTG	58
SA20	AGTCTCTGGCCTTGCAGGTA	GGATCAATGTCCCCAATCAC	59
SA21	ATGGCTGCTCTCAAATGCTT	CTTTTCCTAAACCAGCTGCC	55
SA22	AGGATGCCATGTTGGTTCTC	CCATTGCTAGAAACTCCCCA	57
SA23	GTGCAGCGCAACAACATAAC	GGCCAATAGCTTCCATTCAA	57
SA24	GTCCGTTCTCCTCGCTCTTC	TGTAGGTAGGCAACGGAAGG	61
SA25	CGCATACATCATCATCGTCA	GCCTGGATACGTGAACCACT	55
SA26	TGTATCCCTGCCGTTTCTTC	GAAACTTCCCACTTCGCTCA	57
**B: LTR-IRAP**			
**Primer name**	**Forward**	**Reverse**	**TM**
LTR 1	TGCCACGATCAGCAAGAATCA	TCTCTTGACAATTCACGTGGCT	57
LTR 2	AGTAACTGTAAGCTGACGTGGCT	GGTGTTGTAGAATCTTCCAGACT	53
LTR 3	CCGTTTTGCCGTCTGATCTCT	AATCCACCTCCTCGTGGGAT	58
LTR 4	TGTGGTGCAGTGAACCATTCA	TCGGCTGGAAACCCGAGCTTGC	59
LTR 5	GCTCTCTGGCTGTTATCGGTT	AGGTTGGCCGAACCACGTAA	54
LTR 6	TGCGAATCCACATGGTGATCACA	GGATCGTGATCTAGGAGCCTA	56
LTR 7	TCGTCAATCCGCATGGCTTCCA	GACGTAGGCTAAAAGCCGAACCA	57
LTR 8	GATACCAGGCTCTTACGGGACAC	CAACCGGCGTGCTCTGACTTGT	53
LTR 9	GACTTCGCCCAAACTTTGTGA	GTAGGCGGGGATTGCCGAACCA	55
LTR 10	TCGCCGTTGTTCGTTGAGTGTCT	CGAACCACGTAAAAATCCGCGTG	56
LTR 11	CAGCAACTGCACTGTTCCAGA	TCACTGTGGAGACGATCTTGA	55
LTR 12	TGCGAATCCACATGGTGATCACA	TAGGAGCCTAAATCACTTCA	53
LTR 13	CTCCTAATGGTTCCTAATACCAGACAA	ACCTCTCGAATTGTAGGTCAGG	56
LTR 14	GCAAACCAAGATTGGTGAGGGCA	GCAACCCGTTTTCGTCCAGA	57
**C: LTR-REMAP**			
Primer name		Primer combination	
SA1-REMAP		F 1 REMAP/R AM-SSR10	
SA2-REMAP		F 2 REMAP/R AM-SSR12	
SA3-REMAP		F 3 REMAP/R AM-SSR17	
SA4-REMAP		F 4 REMAP/R AM-SSR25	
SA5-REMAP		F 5 REMAP/R AM-SSR22	
SA6-REMAP		F 6 REMAP/R AM-SSR24	
SA7-REMAP		F 7 REMAP/R AM-SSR23	
SA8-REMAP		F 8 REMAP/R AM-SSR2	
SA9-REMAP		F 9 REMAP/R AM-SSR11	
SA10-REMAP		F 10 REMAP/R AM-SSR14	
SA11-REMAP		F 1 REMAP/R AM-SSR6	
SA12-REMAP		F 2 REMAP/R AM-SSR9	
SA13-REMAP		F 3 REMAP/R AM-SSR11	
SA14-REMAP		F 4 REMAP/R AM-SSR8	
SA15-REMAP		F 5 REMAP/R AM-SSR6	
SA16-REMAP		F 10 REMAP/R AM-SSR12	
SA17-REMAP		F 11 REMAP/R AM-SSR5	
SA18-REMAP		F 12 REMAP/R AM-SSR12	
SA19-REMAP		F 13 REMAP/R AM-SSR16	
SA20-REMAP		F 14 REMAP/R AM-SSR14	

### LTR-IRAP analysis

Fourteen novel LTR-IRAP primers, including *Ty*1/copia-like and *Ty*3/gypsy-like elements, were chosen for this study (Table 2). Primers were designed against the element’s 5′end in the long terminal repeat (LTR) of each retrotransposon. The LTR-IRAP protocol was developed by adaptation of an original method of Kalendar et al. [10]. The amplification programmed consisted of pre-denaturation at 94 °C for 2 min; 35 cycles at 94 °C for 30 s, 60 °C 30 s, ramp + 0.53 °C/s, 72 °C for 90 s, and a final incubation at 72 °C for 10 min. The PCR products were subjected to electrophoresis on a 2% NuSieve® 3:1 agarose gel (Lonza Rockland, Inc.) in 1X TBE buffer stained with ethidium bromide and photographed in BIORAD automated Gel Documentation System (Italy).

### LTR-REMP analysis

Ten primers synthesized from *Ty*-1/copia and *Ty*3-gypsy-like sequences were combined with ten citrus SSR primers performing twenty nine LTR-SSR primer combinations (Table 2). The LTR-REMAP analysis was executed following Kalendar et al. [10]. The thermal cycling was programmed as initial denaturation cycle at 94 °C for 4 min, followed by 35 cycles at 94 °C for 45 s, 55 °C for 40 s, and 72 °C for 1 min for denaturation, annealing and extension, respectively. A final extension step was performed at 72 °C for 10 min. Then, amplification products were separated and visualized following the same procedure described for LTR-IRAP.

### Data scoring and polymorphism analysis

Only reproducible amplicons of each replication will be scored. Consensus profiles were verified based on the presence (1) or absence (0) of amplicons and assembled onto a data matrix. Comparisons of the discriminating capacity, level of polymorphism, and informativeness of each marker system of SSR, LTR-IRAP, and LTR-REMAP were assessed using GenAlEx software [28]. To compare the efficiency of the three markers in citrus species, we estimated the following parameters: (*N*) number of species per group, (Na) number of different alleles, (Na/b) number of alleles or bands, (%*P*) the percentage of polymorphism, (Ne) number of effective alleles, (*I*) Shannon’s index, and (uHe) unbiased expected heterozygosity (Table 3).

**Table 3:** Levels of effective alleles, Shannon’s information index, unbiased expected heterozygosity value, No. of private alleles, and No. of LComm alleles ingenerated by SSR, LTR-IRAP, and LTR-REMAP assays for 20 citrus genotypes

Marker system	Group	*N*	Na/b	%*P*	Na	SE	Ne	SE	*I*	SE	uHe	SE	N. private alleles	N. LComm alleles (≤ 50%)
SSR	Kumquat	1	41	70.83%	1.71	0.09	1.71	0.09	0.49	0.07	0.71	0.09	0.208	0.292
	Acids	6	75	95.83%	3.13	0.26	2.27	0.17	0.87	0.08	0.54	0.04	0.667	0.542
	Grapefruits and pummelos	4	58	79.17%	2.42	0.22	1.93	0.18	0.64	0.09	0.43	0.06	0.208	0.333
	Mandarins	3	54	91.67%	2.25	0.16	1.88	0.15	0.64	0.07	0.49	0.05	0.167	0.250
	Oranges	6	64	95.83%	2.67	0.17	2.21	0.13	0.82	0.06	0.56	0.03	0.083	0.333
LTR-IRAP	Kumquat	1	61	0.00%	0.40	0.04	1.00	0.00	0.00	0.00	0.00	0.00	10	8
	Acids	6	104	60.53%	1.29	0.07	1.31	0.03	0.29	0.02	0.21	0.02	19	21
	Grapefruits and pummelos	4	67	22.37%	0.66	0.07	1.16	0.03	0.13	0.02	0.10	0.02	5	8
	Mandarins	3	84	36.18%	0.91	0.07	1.23	0.03	0.20	0.02	0.16	0.02	6	15
	Oranges	6	89	45.39%	1.04	0.08	1.30	0.03	0.25	0.02	0.18	0.02	5	12
LTR-REMAP	Kumquat	1	100	0.00%	0.33	0.03	1.00	0.00	0.00	0.00	0.00	0.00	15	24
	Acids	6	182	57.00%	1.18	0.06	1.27	0.02	0.27	0.01	0.19	0.01	24	40
	Grapefruits and pummelos	4	152	29.67%	0.80	0.05	1.20	0.02	0.17	0.02	0.13	0.01	15	25
	Mandarins	3	129	25.67%	0.69	0.05	1.17	0.02	0.15	0.01	0.12	0.01	10	14
	Oranges	6	197	59.00%	1.25	0.05	1.32	0.02	0.29	0.02	0.21	0.01	15	51

*N* No. of species per group, *Na/b* No. of alleles or bands, *%P* percentage of polymorphic loci, *Na* No. of different alleles, *Ne* No. of effective alleles, *I* Shannon’s Information Index

### Species diversity and clustering

Principal coordinate analysis (PCoA) will be carried out to display the multidimensional genetic relationship and its partition among varieties [29]. To gain further perspectives on the genetic structure among the citrus germplasm, the Bayesian clustering method to infer the pattern of genetic structure was employed using STRUCTURE 2.2.3 [30, 31]. To estimate the best number of clusters, three independent simulations were achieved per number of sub-groups K (30 runs of K = 1–10). The ideal K number is created on the highest average of the estimated ln probability score that shows the lowest variance for each run. The bar plot of the Structure output was colored according to the K number of sub-groups of the maximum likelihood log with the lowest variation.

## Results

### Level of polymorphism and discriminating power

**Fig. 1:**
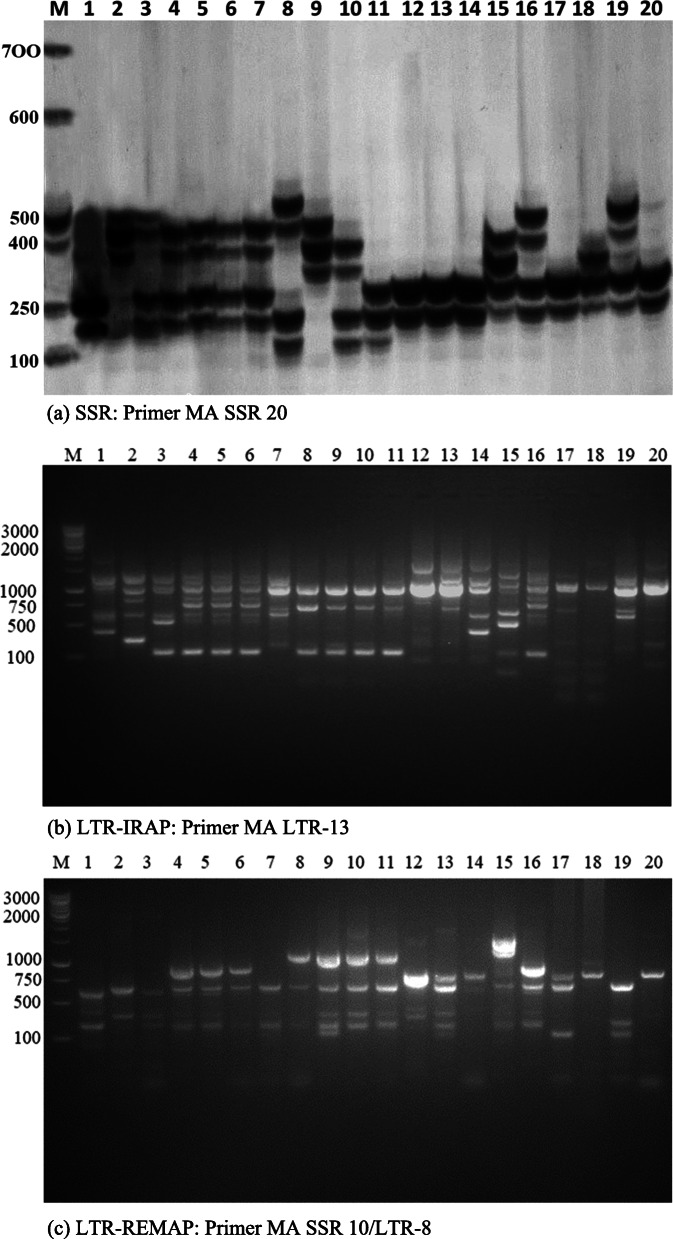
**a** SSR, **b** LTR-IRAP, and **c** LTR-REMAP profiles of 20 citrus genotypes

**Fig. 2:**
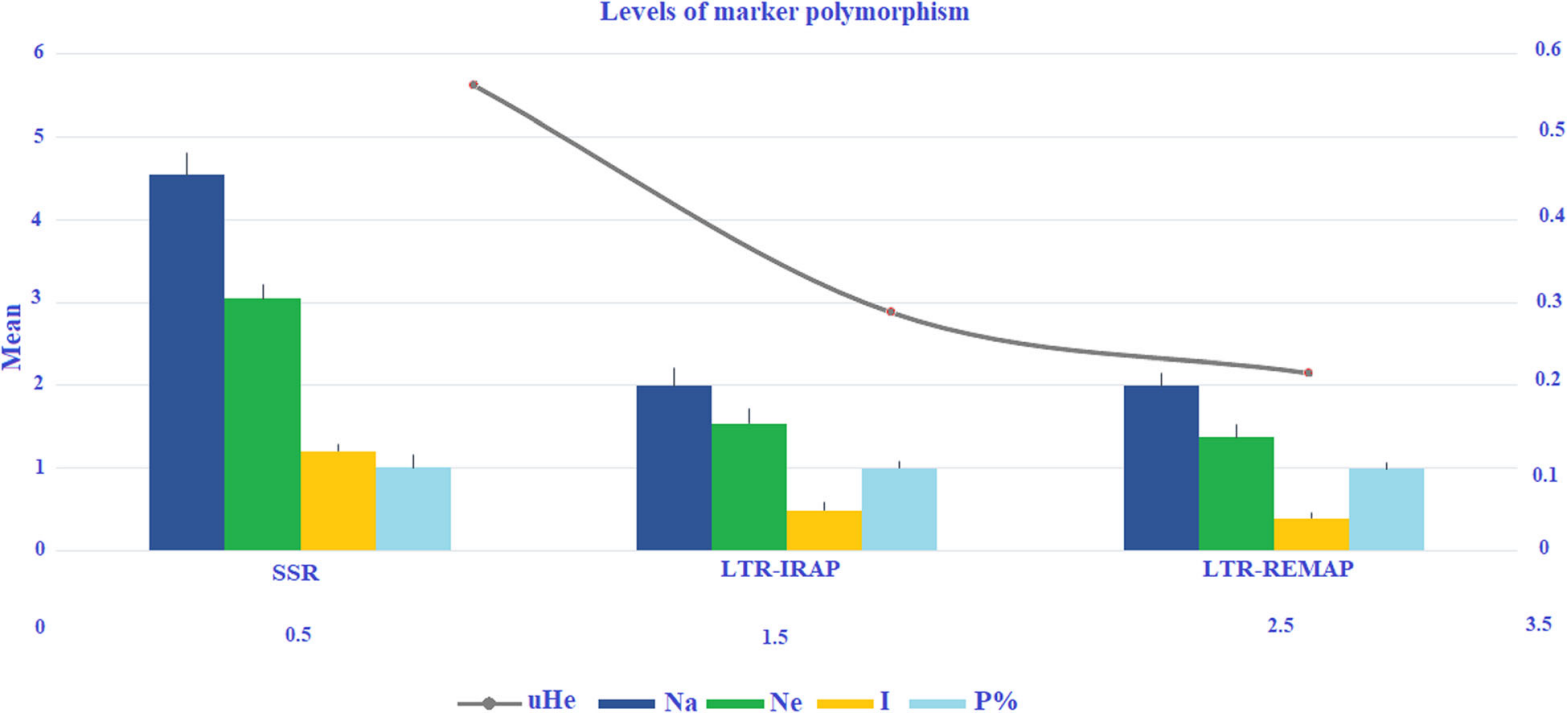
Schematic representation the comparison information obtained of the levels of polymorphism in SSR, LTR-IRAP, and LTR-REMAP markers within 20 genotypes of citrus and its relatives species

In this study, we used a total of 20 genotypes of citrus and its related species, to investigate either SSR, LTR-IRAP, or LTR-REMAP markers were polymorphic sufficient to be suitable for genotype discrimination and breeding programs of citrus. These species were divided into five groups according to the morphological description and fruit characterization as following, acids (six species), grapefruits and pummelos (four species), mandarins (three species), oranges (six species), and a unique kumquat species (Table 1). All marker systems observed turned out to be beneficial tools for detecting polymorphism and assessing genetic diversity in citrus germplasm, while the degree of resolution differed on the applied technique. We primarily tested 70 SSR, 35 LTR-IRAP, and 50 REPAP primers between the five citrus germplasm groups. Among all, 26 SSR, 14 LTR-IRAP, and 20 LTR-REMAP primers provided high polymorphism levels as presented in Table 2 and Fig. 1a–c. Within the three markers system, SSR followed by LTR-IRAP and LTR-REMAP presented diverse polymorphism levels as shown in (Table 3 and Fig. 2). However, in SSR markers, the acid species had superior Na/b, %P, Na, Ne, and I, with mean of 75, 95.83%, and 3.13 ± 0.26, and 2.27 ± 0.17 and 0.87 ± 0.08, respectively. Meanwhile, the lowest Na/b, %*P*, Na, Ne, and *I* appeared only in kumquat species with 41, 70.83, 1.71 ± 0.09, and 1.71 ± 0.09, 0.49 ± 0.07, respectively. In contrast, Kumquat species exhibited the greatest value of uHe with mean of 1.71 ± 0.09. In LTR-IRAP markers, acids species had the higher values in all parameters with mean of 104 for Na/b, 60.53% for %*P*, 1.29 ± 0.07 for Na, 1.31 ± 0.03 for Ne, 0.29 ± 0.02 for *I*, and 0.21 ± 0.02 for uHe, while the grapefruits and pummelos presented the lowest value of Na/b, %*P*, Na, Ne, *I*, and uHe with mean of 67, 22.37%, 0.66 ± 0.07, 1.16 ± 0.03, 0.13 ± 0.02, and 0.10 ± 0.02, respectively. On behalf to the LTR-REMAP markers, oranges showed the high proportions of Na/b, %*P*, Na, Ne, *I*, and uHe with mean of 197, 59%, 1.25 ± 0.05, 1.32 ± 0.02, 0.29 ± 0.02, and 0.21 ± 0.01, respectively. In contrast, mandarins revealed the minority of Na/b, %*P*, Na, Ne, *I*, and uHe with mean of 129, 25.67%, 0.69 ± 0.05, 1.17 ± 0.02, 0.15 ± 0.01, and 0.12 ± 0.01, respectively.

Concerning the behavior of the private alleles (or bands), it correlates positively with the LComm alleles (≤ 50%) across the three markers system (Table 3). Within the species group, acids are the greatest private alleles (in SSR) and banding patterns (in LTR-IRAP and LTR-REMAP), being 0.667, 19, and 24, respectively. In contrast, oranges exhibited the lowest private alleles in SSR with a mean of 0.083, while oranges, grapefruits and pummelos, and mandarins showed slightly lower banding patterns in LTR-REMAP being 15, 15, and 10, respectively. Furthermore, oranges, grapefruits and pummelos, and mandarins recorded the lowermost values of banding patterns in LTR-IRAP being 5, 5, and 6, respectively. Table 4 shows the results for the coefficients correlation (*r*) among the three markers with similarity matrices are presented. Values of *r* were significant correlations were observed when comparing the SSR and LTR-IRAP (0.97) markers and between LTR-IRAP and LTR-REMAP (0.95).

**Table 4:** Coefficients correlation (*r*) among the three markers with similarity matrices for each marker of SSR, LTR-IRAP, and LTR-REMAP markers in 20 citrus genotypes

	SSR	IRAP	REMAP
SSR	1		
IRAP	0.978183	1	
REMAP	0.943912	0.950505	1

### Species diversity and genetic structure

**Fig. 3:**
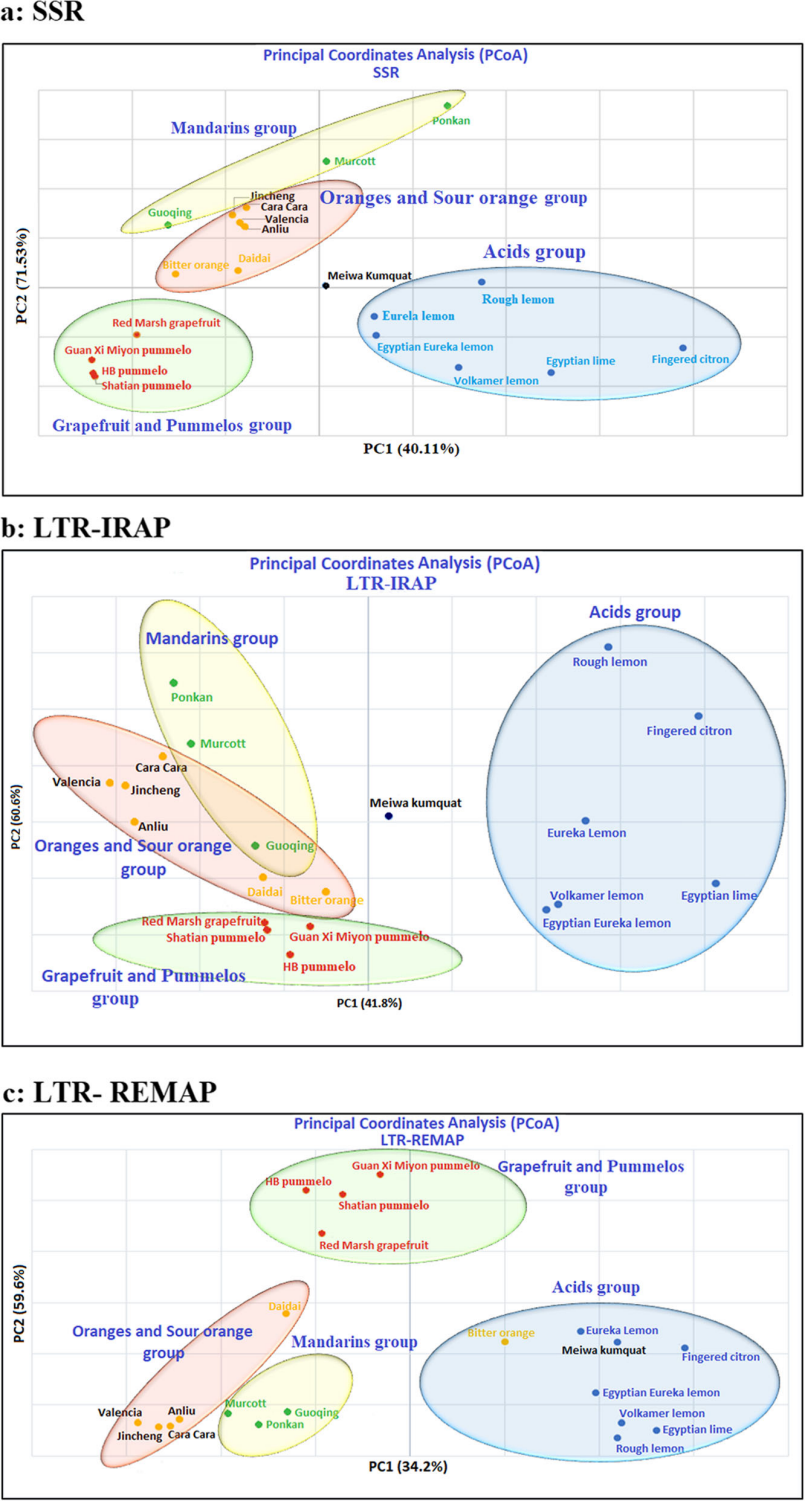
Schematic representation the principal coordinate analysis (PCoA) among 20 genotypes of citrus and its relatives species via **a** SSR, **b** LTR-IRAP, and **c** LTR-REMAP

To further determine the genetic relationships among the citrus species and the resolution of the individual markers, a graphic demonstration of the principal coordinate analysis (PCoA) was constructed to express the results based on data obtained from the SSR, LTR-IRAP, and LTR-REMAP markers. The two-dimensional PCoA plot separated the studied species within the standard four quadrates. The PCoA plot for SSR data revealed 40.11% and 71.53% of the total molecular variation (Fig. 3a). Cluster I compressed all species of acids group, while cluster II assembled all mandarin species in a particular group. Additionally, oranges species were placed together as cluster III; meanwhile, grapefruits and pummelos species were place jointly as cluster IV. The outgroup species, kumquat, was separated individually near the zero values of the axis, while the LTR-IRAP data revealed 41.8% and 60.6% of the total molecular variation (Fig. 3b).

**Fig. 4:**
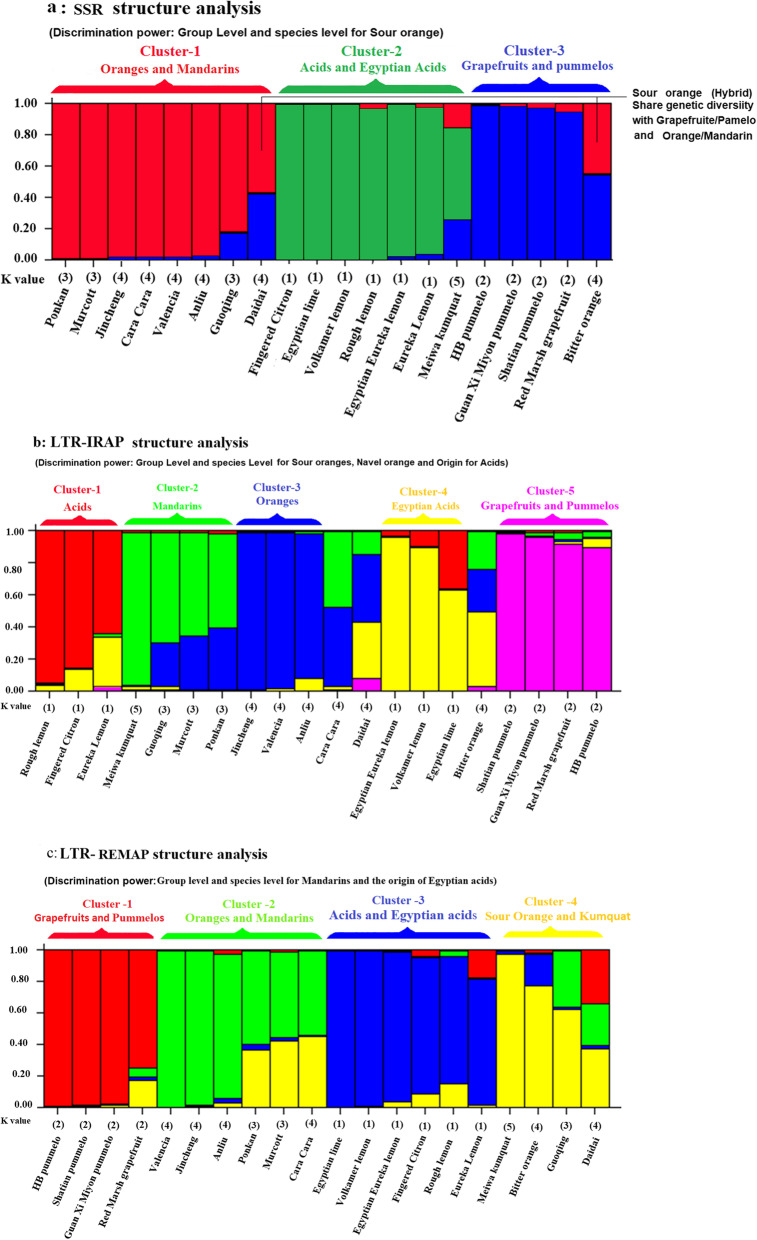
Structure bar plot graph of 20 genotypes of citrus and its relatives species, **a** SSR marker *K* = 3, **b** LTR-IRAP marker *K* = 5, and **c** LTR-REMAP marker *K* = 4. Samples are ordered by group assignment

In comparison with the SSR plot, a clear intersect presented between cluster II (oranges) and cluster III (mandarins) as exposed in guoqing species. However, the LTR-REMAP data revealed 34.2% and 59.6% of the total molecular variation (Fig. 4c). Each species was clustered based on their groups except for bitter orange species clustered with the acids group. Overall, the PCoA for SSR data were strongly distinguishable among the genus citrus that is easily detected and clearly divided into four major separate categories by SSR compared to LTR-IRAP and LTR-REMAP markers.

Concerning to the genetic structure among the citrus species, we used a non-spatial Bayesian clustering method to determine the best number of sub-populations (*K*) based on the highest probability and the lowest variance of each possible number of *K*. The obtained citrus population structure was represented in Fig. 4a–c. The SSR markers’ output results revealed that, the average estimated in probability score and lowest variance (LnP(D)), the most probable sub-population number was *K* = 3. These results representing that the samples are clustered into three main groups and probably originated from three sub-populations (groups) (Fig. 4a). The first group possesses all number of orange and mandarin species. While the second group compresses all acids species, however, group three collected all species of grapefruit and pummelo. Meanwhile, kumquat partially shared to acids group with small a portion of grapefruit/pummelo and orange/mandarin. On the other hand, sour orange (hybrid) shares genetic diversity with grapefruits/pummelo and orange/mandarin.

Concerning to LTR-IRAP marker (Fig. 4b), the samples are clustered into five sub-populations (*K* = 5). The acid species were clearly distinguished within two clustered, not based on their type but on their sampling origin, while the Egyptian acids are gathered with some portions of the other acids and vice versa. The central group composed mainly of sweet orange species, some of which are partly mixed to the mandarin group, while the last group compresses all species of grapefruit/pummelo with very tiny portions of all other groups (acids, mandarin, sweet orange, and Egyptian acids). However, sour orange (Hybrid) share genetic diversity with a small portion of grapefruit/pummelo, while affected with the acids group with equal portions of sweet orange and mandarin, whereas navel orange (hybrid) share genetic diversity with mandarin and sweet orange with a small amount of lemon.

With respect to LTR-REMAP marker (Fig. 4c), the samples are clustered into four sub-populations (*K* = 4). The pummelo species were clearly distinguished within the first clustered while the individual species red marsh grapefruit showed a few mixed portions to kumquat with a weak attachment to orange/mandarin and acids. The second group possesses several attachments, and all sweet orange compress together, while mandarin and navel orange share a high percentage with kumquat, whereas the third group partly shared all species of acids with a small part of grapefruit/pummelo and kumquat. On the other hand, sour orange showed high proportion to kumquat with a mixed portion to acids with a weak attachment to grapefruit/pummelo and orange. However, the hybrid mandarin (guoqing) showed a high percentage of similarity with kumquat while the rest is orange/mandarin with a small acid portion.

## Discussion

Information about the citrus genome and genetic variations present inside and among citrus species and their population’s structure can play a useful role in the effective utilization of citrus breeding. Toward this effort, a variety of molecular marker techniques have been utilized during the past few decades. Mining polymorphisms in the DNA sequence of diverse plants are the principal stage toward the progress and application of molecular markers. These polymorphisms can be a gene, part of a gene, a protein, or a sequence in a non-generation [32]. Presently, several PCR-based systems for marker development exist and have been described in the literature for citrus. However, the progress of molecular DNA markers for genetic analysis has significantly improved our understanding genomes’ structure and performance.

In plants, retrotransposons and microsatellites regions have been extremely successful as evident to their abundance. Their ubiquity in the plant kingdom suggests that they are of very ancient origin [33]. Besides, their abundance has played a significant role in genome evolution and structure [34]. The set of SSR, LTR-IRAP, and LTR-REMAP markers employed were selected based on previous experiences that allowed us to select those markers that amplify a single locus. In this article, co-dominant marker such as SSR, and the dominant markers LTR-IRAP and LTR-REMAP proved to be highly useful tools in discriminating power between the five studied groups of citrus genotypes. Currently, the relatively high values of the effective number of alleles for all the markers used indicate their discrimination power when conducting a large number of specimens. This is very critical for the management of germplasm banks where numerous cultivars need to be identified and correctly [35]. In this revise, SSR provides more effective information on Na/b, %*P*, Na, Ne, and *I* compared to LTR-IRAP and LTR-REMAP, while the high relatively level in number of private alleles and LComm alleles followed the pattern: LTR-REMAP > LTR-IRAP > SSR. This finding suggested that SSR had discrimination power for group level only and species level for sour orange, while LTR-IRAP is able to discriminate group level, species level, and distinguished the origin of acids. Likewise, LTR-REMAP is qualified for assorting the origin for Egyptian acids and species level for mandarins as well group level, probably due to the principle of the LTR-REMAP marker technique. It is the integration of SSR and LTR-IRAP techniques. Many studies confirmed that a LTR-REMAP marker had the greatest discrimination power and many variations at individual loci within citrus species [8, 9]. Compared to other regularly used molecular markers such as SSR, the LTR-REMAP markers are better suited for variation in citrus. A feature of LTR-REMAP is that polymorphisms at various loci are discovered in a particular assay, while SSR usually identified polymorphisms at one locus [36]. Recent evidence of the genome sequence suggests that LTR regions occupied about 28.1 Mb, accounting for 9.74% of the whole genome [20]. This phenomenon is due to the rich presence of *Ty*-1 copia retrotransposons owing to a highly diverse polymorphism between the tested of citrus species [37].

Citrus taxonomy and phylogeny are regularly controversial because of the high diversity of phenotypic characters, their long history of cultivation, and complex reproduction system [20]. Our structure analysis results presented shared ancestry between the root-stocked and domesticated citrus species, suggesting that gene flow has occurred between acids, grapefruit and pummelo, mandarin, orange, and kumquat. The outcome of the current structure analysis is, in our opinion, more representative of the populations. However, the genus *Fortunella* contains the kumquats; it was nested within citrus species, although their morphology is significantly diverse from each other. Several evidence recommended *Fortunella* was the most simple and primitive genus while citrus was in the top stage of evolution because their fruits are edible and important economically [38]. Within this framework, our results synergistically recommend that *Fortunella* has interspecific variations, but it is a single independent group as a genus. Indeed, we are able to tentatively imply forward this theory as *Fortunella* might be less divergent than citrus at the molecular level than observed in morphology, concurred with the previously reported by [8, 37, 39, 40].

In this study, the separation of the three true *C. reticulata, C. medica,* and *C. maxima* in distinct group or subgroup in our LTR-REMAP, SSR, and LTR-IRAP analyses confirms their distinctiveness as the valid basal species of edible citrus. This concept has gained much acceptance and support through previous molecular findings [20, 39, 41, 42].

It is widely believed that sour orange (*C. aurantium* L.) was the most widespread rootstock in Egypt. Evidence suggested that sour oranges are natural hybrids of mandarin and pummel [43, 44]. In this study, bitter orange and daidai considered to be sour orange (*C. aurantium* L.) were nested together with loosely aligned with grapefruit and pummelo. This finding is in agreement with SSR data [45]. Likewise, pummelo is also thought to be a true citrus species [43], which gave rise to sour oranges and grapefruits through hybridization [44]. Indeed, the pummelo genome (*C. maxima*) has played a part in the parentage of many of the cultivars of citrus [45]. This result confirms that pummelo was the maternal parent of *C. sinensis*, *C. aurantium*, *C. paradisi*, and *C. lemon*. Our findings agreed with the previously reported of pummelo (C. *maxima*) (Tanaka classification) [20, 46–48].

The grapefruit (*C. paradisi*) has notified as a natural hybrid between pummelo (*C. maxima*) and sweet orange (*C. sinensis*) [23]. Grapefruit has more significant similarity with pummelo than sweet orange in morphology and chemical constitution, indicating backcross to pummelo. Our data was evolutionarily close grapefruit with pummelo, supporting the viewpoint of a backcross with pummelo. This hypothesis is confirmed by our SSR, LTR-IRAP, and LTR-REMAP data, since the grapefruit accession reveals identity with all pummelo species. Parallel results were obtained by [6, 23, 41, 49–51].

Another striking characteristic, volkamer lemon, appears as one of the excellent control rootstock. Due to their tolerance to biotic and abiotic factors, that is the important purpose for breeding programs. Indeed, it is a more controversial origin [42]. In Egypt, volkamer lemon is the second most promising rootstocks after sour orange (*C. aurantium* L.). Preliminary data supposed that probably originated from mandarin × sour orange [44], or lemon × sour orange [47], or mandarin × citron origin was also recommended [52]. Recently, it is apparently *C. medica* that was the candidate male parent of *C. volkameriana, C. aurantifolia, C. jambhiri,* and Palestine lime [42, 53]. In view of the performance of our results, volkamer lemon is classified with all acid citrus (Egyptian lemon and Egyptian lime) as the taxon with which it seems to be highly strongly associated. Herein, our results following the recent theory of nuclear and cytoplasmic data show that citron, lemon, rough lemon, lime, and volkamer lemon were more significant affiliation compare the other citrus and related genera [23, 42, 48].

Several earlier workers hypothesized *C. limon* to be of complex hybrid origin involving two parents: citron and lime [16, 43, 54] or citron and sour orange [47, 55] or lime and sour orange [56, 57]. As seen in our results, *C. limon* (Eureka lemon and Egyptian Eureka lemon) reveals that it has a close affinity to *C. aurantifolia* (Egyptian lime), *C. medica* (Fingered citron), *C. jambhiri* (Rough lemon), and *C. volkameriana* (Volkamer lemon) proves their potential mixture origin, as previously observed by the cytogenetic, phylogenetic, and genomic analysis [20, 52, 58, 59].

The citron mitotype contained only *C. medica* and is considered a real male parent in citrus breeding. Indeed, using the nuclear and cytoplasmic analysis, [47] revealed that citron was the male parent of Mexican lime, Palestine sweet lime, rangpur lime, volkamer lemon, and rough lemon. This trend was supported by earlier cytoplasmic and nuclear data of Curk et al. [42] who point to *C. medica* as likely to be the directly male parent of lemon and lime; this is due to the shared genomic structure. Our data confirm this hypothesis since the citron mitotype show identity with lime and lemon.

Our data suppose a theory as *C. reticulata* (mandarins) was evolutionarily close with *C. sinensis* (sweet orange), emphasize that *C. reticulata* was shared between mandarins and sweet orange, consistent with the assumptions of Barrett and Rhodes [44] and Nicolosi et al. [47]. Many studies confirmed this opinion as *C. reticulata* and *C. sinensis* were clear signs of the narrow genetic basis and indicated that sweet orange and citron may be female and male parents, respectively [23, 41, 60]. This trend is supported by the recent reclassification of citrus origin [20], confirming that among *C. reticulata* and *C. sinensis*, they found a vast assembly of linkage that endorses the domestication of these groups.

## Conclusion

In plant genomes, retrotransposons, and microsatellite elements represent the main abundance component of the structural evolution, differing greatly in copy number within the genome. To facilitate such purposes, here, we report a detailed overview of genetic structure and PCoA analysis based on SSR, LTR-IRAP, and LTR-REMAP approach can capture the genetic relationships and evolution within the genus citrus and related species. Herein, the result of the co-dominant SSR marker can differentiate within the species level of sour orange and has able to be identifying the group level of citrus. However, the dominant LTR-REMAP marker was more sensitive in most discrimination parameter than SSR and LTR-IRAP. It could be classifying the origin of Egyptian acids, species level for mandarins, and the group level of citrus, due to it is the integration of SSR and LTR-IRAP techniques. Currently, our finding of the LTR-REMAP structure analysis support the monophyletic nature of the citrus species; able to provide unambiguous identification and disposition of true species and related hybrids like lemon, lime, citron, sour orange, grapefruit, mandarin, sweet orange, pummelo, and fortunella; and resulted in their placement in individual or overlap groups. Interestingly, we observed a thorny of the two Egyptian rootstocks mitotypes, sour orange, and volkamer lemon, supporting the viewpoint that citron was the candidate male parent, while grapefruit/pummelo and mandarin acted acts as the female parent of sour oranges and volkamer lemon. This article offer a useful and potential additional knowledge for breeding programs and conservation approaches as an essential step toward understanding the interspecific admixture and the inferred structure origins of Egyptian citrus rootstock and acid cultivars.

## Data Availability

The datasets used and/or analyzed during the current study available from the corresponding author on reasonable request.

## Abbreviations

SSR: Simple sequence repeats
LTR: Long terminal repeats
RT: Retrotransposons
IRAP: Inter-retrotransposon amplified polymorphism
REMAP: Retrotransposon-microsatellite amplified polymorphism
PCoA: Principal coordinates analysis
